# The Impact of Enteral Nutrition Type, Volume, and Time of Introduction on the Risk of Growth Failure and Bronchopulmonary Dysplasia in Preterm Infants

**DOI:** 10.3390/nu18020283

**Published:** 2026-01-16

**Authors:** Karen D. Hendricks-Muñoz, Miheret S. Yitayew, Nayef Chahin, Allison Williams, Jie Xu, Adeola Abdulkadir, Bemnet Alemayehu, Judith A. Voynow

**Affiliations:** 1 Virginia Commonwealth University School of Medicine, Richmond, VA 23298, USA; miheret.yitayew@vcuhealth.org (M.S.Y.); nayef.chahin@vcuhealth.org (N.C.); allison.williams@vcuhealth.org (A.W.); jie.xu@vcuhealth.org (J.X.); bemnet.alemayehu@vcuhealth.org (B.A.); judith.voynow@vcuhealth.org (J.A.V.); 2 Children’s Hospital of Richmond at VCU, Richmond, VA 23298, USA; 3 Eastern Virginia Medical School, Norfolk, VA 23501, USA; abdulka@odu.edu

**Keywords:** mother’s own milk, pooled pasteurized donor human milk, preterm infant, growth, bronchopulmonary dysplasia

## Abstract

**Background/Objectives**: Greater than 50% of surviving very preterm infants are affected by postnatal growth failure and are at high risk of associated development of bronchopulmonary dysplasia (BPD). Given the influence of enteral feeding on growth failure, we aimed to determine the impact of type, volume, and time of introduction of enteral feeds on mitigating the risk of postnatal growth failure and BPD risk. **Methods**: This was a retrospective chart review of mothers’ own milk (MOM), pooled pasteurized donor human milk (PDHM) feeding, postnatal growth, and BPD severity in preterm infants <33 weeks of gestation admitted to the Children’s Hospital of Richmond at VCU neonatal intensive care unit between 2021 and 2024. Statistical analysis included linear regression with moderation analysis using the Hayes Process model, chi-square tests, linear and multinomial logistic regression, with *p*-value < 0.05 considered significant. **Results**: After controlling for the percentage of MOM received at 34 weeks corrected gestational age (cGA), greater severity of BPD was associated with lower infant weight and growth failure, *p* < 0.001. Early introduction of MOM (3 days of life) and greater volume of MOM showed better linear growth and decreased risk of severe BPD, respectively (*p* < 0.001). **Conclusions**: Provision of MOM to preterm infants within 3 days of life was associated with a moderation of the relationship between gestational age and growth velocity, with improved growth velocity trajectory. Preterm infants who received a greater volume of MOM through 34 weeks cGA experienced less severe BPD compared to those fed higher volumes of PDHM. As the incidence of growth failure paralleled the incidence of BPD severity, identification of key MOM components becomes important to address and augment the value of PDHM in the management of preterm infants.

## 1. Introduction

Very preterm infants (vPT) <33 weeks gestation, account for 52% of infant deaths, with >50% affected by postnatal growth failure [1,2]. Growth failure during infancy is a major global problem with associated adverse effects on long-term infant health, including lung and neurodevelopment [3,4]. Mother’s own milk (MOM) is a nutrient source that not only influences preterm infant mortality but also attenuates morbidities from necrotizing enterocolitis, chronic lung disease, and adverse neurodevelopment [5,6,7,8]. Chronic Lung Disease during infancy or bronchopulmonary dysplasia (BPD), a major global problem with life-long morbidities, is a multifactorial disease of disrupted alveolarization and pulmonary vascular development, and impaired lung growth [9,10,11,12]. BPD remains the most common long-term complication of prematurity in those infants < 32 weeks of gestation, which affects not only the respiratory system but also overall growth and development in preterm survivors beyond the neonatal period, extending into childhood and adolescence [12,13,14]. Breastmilk appears to offer lung and immune protective benefits against BPD, allergies, and later childhood asthma [15,16,17,18,19,20,21].

In addition to its protective role against BPD, MOM has been shown to positively impact postnatal growth compared to pasteurized donor human milk (PDHM), often used as a substitute or bridge for feeding the PT infant [22,23,24]. Lund et al. observed that increased intake of MOM compared to PDHM was associated with improved postnatal weight gain and head circumference growth in extremely preterm infants [25].

While human milk is a cornerstone of neonatal nutrition and the nutritional components of human milk have been well described and known to enhance healthy immune development, when a mother’s own milk (MOM) is unavailable, pasteurized donor human milk (PDHM) is commonly used. While PDHM confers protection against necrotizing enterocolitis, it differs substantially from MOM in bioactive composition, and its impact on lung development and growth remains incompletely understood [26,27,28,29,30,31]. Additionally, the influence of early milk colostrum and transitional MOM composition is unique compared to mature PDHM, with limited understanding of this influence on growth or BPD development [32,33,34,35]. Thus, we hypothesized that early (within the first week of life) and sustained exposure to MOM favorably modifies the effects of gestational immaturity and early illness severity (SNAPPE-II) on infant postnatal growth velocity trajectories and reduces the severity of bronchopulmonary dysplasia that would align with growth impairment [36].

Given the influence of breast milk on growth failure and BPD, this study aimed to address a critical knowledge gap by defining how timing and cumulative dose of MOM as compared to PDHM modify the effects of gestational immaturity and illness severity on postnatal growth and BPD, as well as identify any association of BPD risk with growth.

## 2. Materials and Methods

### 2.1. Study Design and Participants

This study was conducted at the Children’s Hospital of Richmond at Virginia Commonwealth University and involved a retrospective analysis of a cohort from a prospective longitudinal observational study. The cohort included infants born at less than 33 weeks of gestation who were enrolled in an Institutional Review Board-approved study (HM20022431). Preterm infants under 33 weeks of gestation and their mothers, regardless of their gender, race, or ethnicity, were included. Exclusions were made for infants with known chromosomal anomalies or major birth defects, or those without written consent.

Infants received feeding according to protocol, initiated upon the receipt of the mother’s milk or, at the mother’s discretion, from PDHM. The volume was advanced based on gestational age, birth weight, and feeding tolerance. Mother’s own milk was collected and stored in containers for immediate use or frozen until provided to the infant, with administration conducted in the order the milk was received. Pasteurized donor human milk was sourced from the King’s Daughters Milk Bank in Norfolk, VA, USA, which operates under the accreditation of the Human Milk Banking Association of North America (HMBANA). All PDHM was barcoded, cultured negatively, and had documented fat, protein, carbohydrates, total solids, and energy.

### 2.2. Clinical Data and Outcomes

Clinical and demographic data were acquired using electronic medical records, capturing information on gender, maternal race and health conditions, gestational age (GA), birthweight (BW), and the type of enteral feeding (MOM or PDHM), as well as feeding volume and timing of introduction. Growth failure was defined as an infant’s weight below the 10th and 3rd percentiles at 34 weeks corrected GA, based on the Fenton infant growth scale [37]. BPD was documented and defined according to the classification established by Jensen et al. This classification assesses disease severity based on the mode of respiratory support received at 36 weeks postmenstrual age, regardless of the use of supplemental oxygen. At 36 weeks corrected GA, BPD severity is categorized as follows: Grade 1 requires ≤2 L/min nasal cannula (classified as mild), Grade 2 involves >2 L/min nasal cannula or non-invasive ventilation (classified as moderate), and Grade 3 includes infants requiring invasive mechanical ventilation (classified as severe) [11]. The severity of health illness was evaluated using the SNAPPE-II [36].

### 2.3. Statistical Analysis

Subject characteristics were summarized using frequencies and percentages or means and standard deviations. The statistical analysis employed linear regression with moderation analysis, utilizing the Hayes PROCESS model, adjusted for infant health severity, as well as Pearson correlations. Sample size and power calculations were performed to detect a relationship at 80% power, establishing a minimum sample size of 29 per group, resulting in a total of 58 mother–infant pairs needed to assess the relationship between growth restriction, diet, and BPD risk. All analyses were conducted with significance defined at a *p*-value of <0.05, using SAS Version 9.4 Statistical Software (SAS Institute, Cary, NC, USA).

Directed acyclic graphs (DAGs) were constructed to guide covariate selection using the DAGitty tool v3.1 (https://dagitty.net/dags.html, 23 December 2025) [38,39]. Based on these diagrams, gestational age and SNAPPE-II score were identified as the minimal sufficient adjustment set, capturing baseline immaturity and illness severity that influence both feeding exposure and clinical outcomes. Variables occurring downstream of milk exposure, such as respiratory support or feeding advancement, were not adjusted to avoid overadjustment bias.

## 3. Results

### 3.1. Covariate Selection with Directed Acyclic Graph Assessment

**Figure 1:**
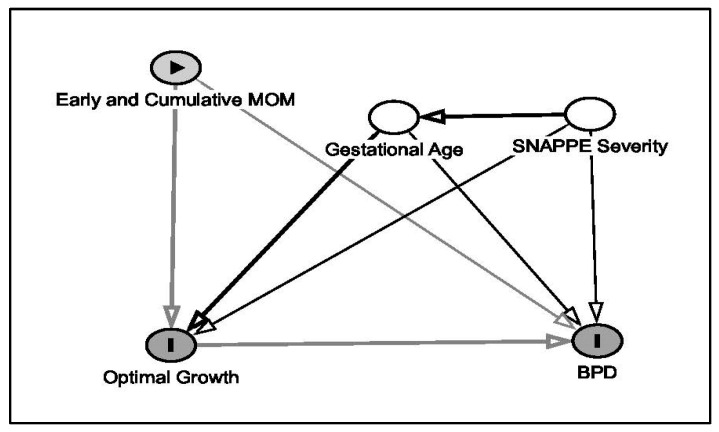
Directed acyclic graph (DAG) of analytic strategy. A directed acyclic graph characterizes the analytical strategy of the study where optimal growth influences BPD and optimal growth and BPD are independently influenced by early and cumulative MOM.

Using directed acyclic graphs (DAGs) to guide covariate selection and analytic strategy, gestational age and SNAPPE-II score were identified as the minimal sufficient adjustment set, capturing baseline immaturity and illness severity that influence both milk exposure and clinical outcomes [38,39]. By avoiding adjustment for downstream mediators such as respiratory support or feeding advancement, we minimized overadjustment bias and preserved the interpretability of observed associations (Figure 1).

### 3.2. Infant Population Characteristics

There were 100 premature infants included in this analysis with a mean gestational age of 27.1 ± 1.5 weeks and a mean birth weight of 1151 ± 441 gms. Racial and ethnic composition included 37% White, 46% Black, and 6% Asian or Other categories, with 11% of the population of Hispanic ethnicity, see Table 1.

### 3.3. Infant Growth Failure and BPD Association

The characteristics of the infants with BPD are shown in Table 2. There was a total of 39 (39%) of infants with BPD, of which 21 (21%) were mild BPD and 18 (18%) were considered moderate/severe BPD. The mean gestational age of the infants with BPD was significantly younger and smaller than that of those who did not develop BPD. There were no significant racial or ethnic differences among the infants with BPD or with later growth failure. There were 41 infants (41%) with growth failure at 34 weeks cGA by weight of <10th percentile and 18 (18%) at <3rd percentile, of which there were greater association of BPD with infants with growth failure at the <10th percentile as well as at the <3rd percentile, Table 2.

### 3.4. Influence of MOM and PDHM on Growth Velocity

Moderations were conducted to examine whether early nutrition type (MOM vs. PHDM) moderated the risk of gestational age on growth velocity.

The first moderation examined MOM in the first three days as a moderator of the relationship between gestational age and growth velocity. The overall regression model was statistically significant, F (3, 179) = 8.41, *p* < 0.001, indicating that gestational age and the mother’s own milk explained 12.35% of the variance in growth velocity (R^2^ = 0.123). Gestational age was a significant negative predictor of growth velocity (b = −0.86, SE = 0.24, t = −3.66, *p* < 0.001, 95% CI [−1.32, −0.40]), indicating that lower gestational age was associated with lower growth velocity. Mothers’ own milk was not a significant predictor of growth velocity (b = −3.08, SE = 8.88, t = −0.35, *p* = 0.729). Importantly, the interaction between gestational age and mother’s own milk was significant (b = 0.41, SE = 0.23, t = 0.46, *p* = 0.046), which indicates that while mother’s own milk alone does not predict growth velocity, mother’s own milk does significantly attenuate the risk conferred by lower gestational age. The change in explained variance due to the interaction term was negligible (ΔR^2^ = 0.123), and the interaction significantly improved model fit.

**Figure 2:**
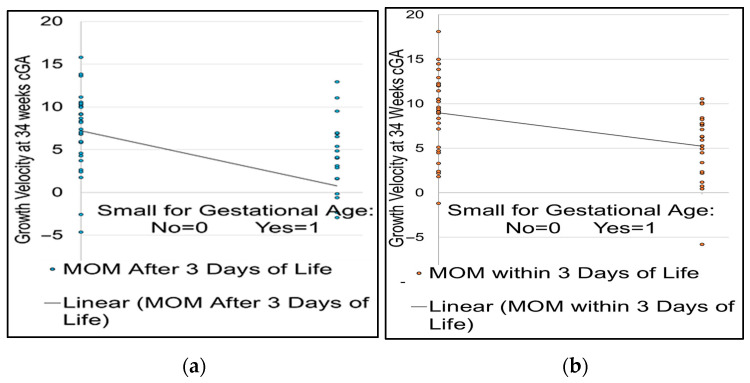
The Impact of Timing of MOM on Growth Velocity. Adjusted for infant health severity using the SNAPPE-II score [36], the figure demonstrates the influence of MOM (**a**) after 3 days and (**b**) within 3 days of life on the moderation of the relationship between GA and growth velocity, including infants at growth failure risk.

The second moderation, which examined whether initiation of PDHM in the first 3 days moderated the risk between gestational age and growth velocity, was not significant. Subsequent moderations examining the moderating influence of MOM and PHDM after day of life 4 on the relationship between gestational age and growth were both not significant, as shown in Figure 2.

### 3.5. Influence of Volume of MOM and PDHM on Risk of BPD

**Figure 3:**
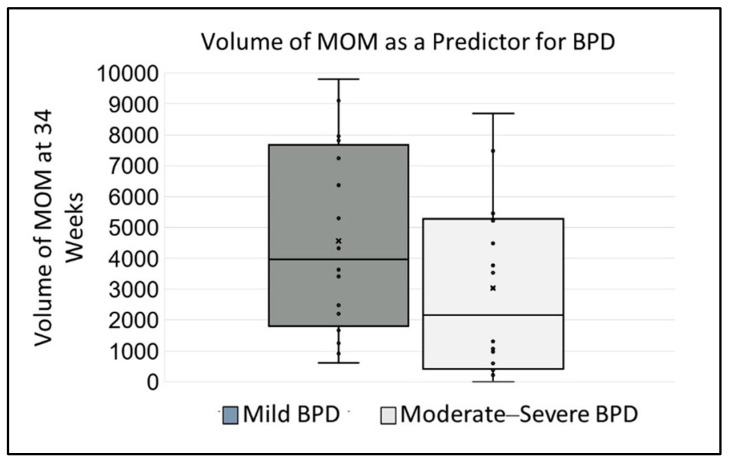
BPD Severity Impacted by Volume of MOM. After controlling for the percentage of MOM at 34 weeks cGA, a greater volume of MOM was associated with a lower severity of BPD.

Using Pearson correlations, we investigated the association of volume of MOM and PDHM on the influence of BPD severity risk. We noted that there was greater severity of BPD associated with lower volume of MOM, r = 0.156, *p* < 001, as shown in Figure 3. This association was not identified for PDHM, r = −0.031, *p* = 0.663.

### 3.6. Association of Growth Failure and Risk of BPD

**Figure 4:**
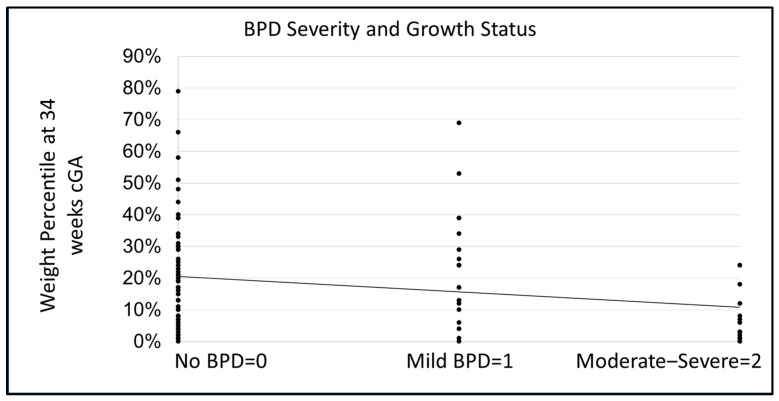
The Influence of Growth Status on BPD Severity. After controlling for health severity using the SNAPPE-II score [36] and % MOM at 34 weeks, a greater severity of BPD was associated with a lower infant weight and growth failure at 34 weeks cGA.

We aimed to assess the association of growth failure and risk of BPD. After controlling for the % volume of MOM and PDHM at 34 weeks, we identified that greater severity of BPD was associated with lower infant weight and growth failure at 34 weeks cGA. Infant weight at 34 weeks was significantly correlated with BPD severity, such that more severe BPD was associated with lower infant weight at 34 weeks, *p* < 0.001, r(125) = −0.332, Figure 4.

## 4. Discussion

Human milk feeding in preterm infants improves feeding tolerance, supports gut health, and is associated with a reduction in the severity of bronchopulmonary dysplasia (BPD) [17,19,22,40,41]. Although human milk is considered the optimal source of nutrition, it does not fully meet the nutritional needs of the very preterm infant, necessitating the addition of micronutrient supplementation. When mother’s own milk (MOM) is not available, pooled pasteurized donor human milk (PDHM) from an accredited human milk bank is recommended [42,43]. However, studies have demonstrated greater postnatal growth restriction in infants fed PDHM compared to MOM [22,23,24]. Additionally, MOM remains the optimal nutritional source associated with a reduction in mortality and morbidities related to necrotizing enterocolitis, chronic lung disease, and adverse neurodevelopment owing to its immune-supporting composition that optimizes gut microbiome, intestinal integrity, and immune development [7,8,9,10,39,44,45,46].

In this study, we observed that exposure to MOM and not PDHM within the first three days of postnatal life was associated with greater growth velocity and attenuation of postnatal growth failure. Furthermore, the cumulative volume of MOM received during hospitalization was significantly associated with reduced BPD severity, while BPD risk was correlated with the degree of postnatal growth failure. These findings continue to support the preferential use of MOM utilization over PDHM in this high-risk population. Our findings differ from those of Avila-Alvarez et al., who reported no significant difference in BPD incidence among premature infants based on whether initial feedings consisted of MOM or PDHM [39]. Importantly, our study incorporated both timing and volume of MOM compared to PDHM, accounting for loss of early colostrum and transitional milk exposure, replete with bioactive components, as compared to substitution with mature PDHM as the initial feeding.

Our study is novel in identifying a relationship between postnatal growth failure and BPD severity. By linking milk exposure to both growth trajectories and pulmonary outcomes, this work bridges traditionally siloed domains of neonatal nutrition and respiratory medicine. Prior studies have demonstrated the nutritional benefits of human milk for very preterm infants and its potential protective role against BPD, allergic disease, and later childhood asthma [5,6,7,17,19,20,22,23,46,47]. Consistent with our findings, human milk feeding has repeatedly been associated with reduced BPD risk, though variability exists in reported effects of MOM versus PDHM on growth and BPD severity [26,46,48,49,50,51]. A 2019 meta-analysis demonstrated that exclusive feeding with unpasteurized MOM was associated with a significant reduction in BPD risk (RR 0.74, 95% CI 0.57–0.96) [7]. However, that analysis of 15 studies did not demonstrate a significant reduction in BPD oxygen requirement when infants were fed PDHM alone, with benefit observed primarily when MOM was supplemented with PDHM [7]. Similarly, a study comparing raw versus pasteurized MOM identified a significantly lower risk of BPD with raw MOM after adjustment for confounders (OR 0.40, 95% CI 0.27–0.67; *p* < 0.001) [52]. Another study reported that PDHM was associated with poorer weight gain and lower neurodevelopmental outcomes compared with MOM, without a significant difference in BPD risk; however, that study was not powered to assess BPD severity [53]. Collectively, these findings suggest that pasteurization and the loss of key bioactive, lipid, anti-inflammatory, and immune-modulating components in PDHM may contribute to observed differences in outcomes [8,53].

Our study had limitations that should be taken into account when interpreting these results. This was a single-center retrospective design study of a prospective cohort with PDHM sourced from one center that may limit the generalization of the results to other units with different populations or clinical practices of enteral feeding or management of BPD. This limitation is somewhat offset by the standard management of the cohort within the same neonatal unit and with guidelines in feeding and clinical care, and respiratory management of infants at risk of BPD. Additionally, while causality cannot be established in an observational study, we strengthened causal interpretability through causal modeling using gestational age and SNAPPE-II covariates, longitudinal data on the volume or proportion of MOM or PDHM received by the infants, as well as the time and initiation of each feeding type during hospital admission. This design is important as infants can receive some PDHM during their course in the NICU, and it was useful to unmask the differences between the PDHM and MOM groups to identify the impact of type, timing, and volume of feeding on health outcomes. These approaches reduce bias and support bio-logic plausibility without overstating causation. Rather than treating human milk as a uniform exposure, we explicitly test whether early MOM vs. PDHM exposure modifies the relationship between gestational age, growth, and BPD severity.

Our results underscore the importance of early-life exposure to MOM in potentially shaping growth trajectories and BPD development in the very preterm infant. Because not all infants have access to MOM for a variety of reasons, it is critical to identify the specific growth-promoting and BPD-modulating components of human milk and make them available to all infants when MOM is not feasible. Components absent or diminished in PDHM, such as microbiota, nutritional lipids, and bioactive factors, may influence both the establishment of the infant’s gut homeostasis and provide modulators of pulmonary immune and growth pathways. Given that nutrition is one of the few modifiable factors that clinicians can leverage to influence postnatal growth failure and risk of BPD, defining key compositional elements of MOM that mitigate BPD severity and growth failure is essential to advance precision-based personalized neonatal care of very preterm infants in the NICU.

Very preterm infants, 22–32 weeks of gestation, representing approximately 1.6% of all US live births, account for 52% of infant deaths, with more than 50% affected by extrauterine or postnatal growth failure [1,2,10,45]. For these infants, MOM or PDHM remains the preferred nutritional source to reduce mortality and morbidities, including necrotizing enterocolitis, chronic lung disease, and adverse neurodevelopment [5,6,7]. Our findings support the early implementation of MOM, as compared to PDHM, not only as an effective dietary strategy to promote optimal infant growth patterns but also as a means to reduce the risk of BPD and its associated long-term health consequences in very preterm infants.

## 5. Conclusions

Introduction of MOM within 3 days of life was associated with a moderation of the relationship between gestational age and growth velocity, and these results were associated with improved growth velocity trajectory in very preterm infants. Preterm infants who received a greater cumulative volume of MOM through 34 weeks corrected gestational age experienced less severe BPD compared to those fed PDHM. Given the identified association between postnatal growth failure and BPD severity, future research should focus on the identification of key MOM components responsible for these protective effects and on addressing the variability in outcomes associated with PDHM.

## Figures and Tables

**Table 1:** Characteristics of the infant overall population.

Characteristics	
Gestational Age (weeks) ± SD	27.1 ± 1.5
Birthweight (grams) ± SD	1151 ± 441
Sex—Female n (%)	55 (55)
Sex—Male n (%)	45 (45)
Race and Ethnicity—n (%)	
White	37 (37)
Black	46 (46)
Asian/Other	6 (6)
Hispanic	11(11)

**Table 2:** Characteristics of the infant BPD population.

Characteristics	No BPD n = 61	Mild BPD n = 21	Moderate–Severe BPD n = 18
Gestational Age (weeks) ± SD	29.1 ± 1.8	26.1 ± 1.9	27.1 ± 2.8
Birthweight (grams) ± SD	1542 ± 323	1109 ± 296	803 ± 230
Growth Failure <10% tle n (%)	23 (38)	4 (19)	14 (78)
Growth Failure <3% tle n (%)	10 (16)	2 (10)	6 (33)
Sex—Female n (%)	30 (50)	14 (67)	11 (61)
Sex—Male n (%)	31 (50)	7 (33)	7 (39)
Race and Ethnicity—n (%)			
White	29 (47)	9 (43)	8 (44)
Black	20 (33)	10 (48)	7 (39)
Asian/Other/Hispanic	12 (20)	2 (9)	3 (17)

## Data Availability

No new data were created, and data are unavailable due to privacy or ethical restrictions.

## Abbreviations

The following abbreviations are used in this manuscript:

MOM	Mother’s own milk
PDHM	Pasteurized Donor Human Milk
BPD	Bronchopulmonary dysplasia

## References

[1] Barfield W.D. (2018). Public Health Implications of Very Preterm Birth. Clin. Perinatol..

[2] Cooke R.J., Ainsworth S.B., Fenton A.C. (2004). Postnatal growth retardation: A universal problem in preterm infants. Arch. Dis. Child. Fetal Neonatal Ed..

[3] Shah P.S., Wong K.Y., Merko S., Bishara R., Dunn M., Asztalos E., Darling P.B. (2006). Postnatal growth failure in preterm infants: Ascertainment and relation to long-term outcome. J. Perinat. Med..

[4] Benjamin-Chung J., Mertens A., Colford J.M., Hubbard A.E., van der Laan M.J., Coyle J., Sofrygin O., Cai W., Nguyen A., Pokpongkiat N.N. (2023). Early-childhood linear growth faltering in low- and middle-income countries. Nature.

[5] Rocha G., Guimaraes H., Pereira-da-Silva L. (2021). The Role of Nutrition in the Prevention and Management of Bronchopulmonary Dysplasia: A Literature Review and Clinical Approach. Int. J. Environ. Res. Public Health.

[6] Spiegler J., Preuß M., Gebauer C., Bendiks M., Herting E., Göpel W., German Neonatal Network (GNN) (2016). Does Breastmilk Influence the Development of Bronchopulmonary Dysplasia?. J. Pediatr..

[7] Villamor-Martinez E., Pierro M., Cavallaro G., Mosca F., Kramer B.W., Villamor E. (2018). Donor Human Milk Protects against Bronchopulmonary Dysplasia: A Systematic Review and Meta-Analysis. Nutrients.

[8] Furman L., Taylor G., Minich N., Hack M. (2003). The effect of maternal milk on neonatal morbidity of very low-birth-weight infants. Arch. Pediatr. Adolesc. Med..

[9] Bancalari E., del Moral T. (2001). Bronchopulmonary dysplasia and surfactant. Biol. Neonate.

[10] Jobe A.H., Bancalari E. (2001). Bronchopulmonary dysplasia. Am. J. Respir. Crit. Care Med..

[11] Jensen E.A., Dysart K., Gantz M.G., McDonald S., Bamat N.A., Keszler M., Kirpalani H., Laughon M.M., Poindexter B.B., Duncan A.F. (2019). The Diagnosis of Bronchopulmonary Dysplasia in Very Preterm Infants. An Evidence-based Approach. Am. J. Respir. Crit. Care Med..

[12] Islam J.Y., Keller R.L., Aschner J.L., Hartert T.V., Moore P.E. (2015). Understanding the Short- and Long-Term Respiratory Outcomes of Prematurity and Bronchopulmonary Dysplasia. Am. J. Respir. Crit. Care Med..

[13] Piersigilli F., Bhandari V. (2020). Metabolomics of bronchopulmonary dysplasia. Clin. Chim. Acta.

[14] Higgins R.D., Jobe A.H., Koso-Thomas M., Bancalari E., Viscardi R.M., Hartert T.V., Ryan R.M., Kallapur S.G., Steinhorn R.H., Konduri G.G. (2018). Bronchopulmonary Dysplasia: Executive Summary of a Workshop. J. Pediatr..

[15] Hicks S.D., Beheshti R., Chandran D., Warren K., Confair A. (2022). Infant consumption of microRNA miR-375 in human milk lipids is associated with protection from atopy. Am. J. Clin. Nutr..

[16] Kho A.T., McGeachie M.J., Moore K.G., Sylvia J.M., Weiss S.T., Tantisira K.G. (2018). Circulating microRNAs and prediction of asthma exacerbation in childhood asthma. Respir. Res..

[17] Fonseca L.T., Senna D.C., Silveira R.C., Procianoy R.S. (2017). Association between Breast Milk and Bronchopulmonary Dysplasia: A Single Center Observational Study. Am. J. Perinatol..

[18] Merino-Hernandez A., Palacios-Bermejo A., Ramos-Navarro C., Caballero-Martin S., Gonzalez-Pacheco N., Rodriguez-Corrales E., Sanchez-Gomez de Orgaz M.C., Sanchez-Luna M. (2024). Effect of Donated Premature Milk in the Prevention of Bronchopulmonary Dysplasia. Nutrients.

[19] Siddiqui A., Voynow J., Chahin N., Williams A., Xu J., Chavez D., Carroll L., Hendricks-Munoz K.D. (2025). Greater and Earlier Exposure of Mother’s Own Milk Compared to Donor Human Milk Moderates Risk and Severity of Bronchopulmonary Dysplasia. Breastfeed. Med..

[20] Villamor-Martinez E., Pierro M., Cavallaro G., Mosca F., Villamor E. (2019). Mother’s Own Milk and Bronchopulmonary Dysplasia: A Systematic Review and Meta-Analysis. Front. Pediatr..

[21] Zhu Y., Chen X., Zhu J., Jiang C., Yu Z., Su A. (2022). Effect of First Mother’s Own Milk Feeding Time on the Risk of Moderate and Severe Bronchopulmonary Dysplasia in Infants with Very Low Birth Weight. Front. Pediatr..

[22] Montjaux-Regis N., Cristini C., Arnaud C., Glorieux I., Vanpee M., Casper C. (2011). Improved growth of preterm infants receiving mother’s own raw milk compared with pasteurized donor milk. Acta Paediatr..

[23] Colaizy T.T., Carlson S., Saftlas A.F., Morriss F.H. (2012). Growth in VLBW infants fed predominantly fortified maternal and donor human milk diets: A retrospective cohort study. BMC Pediatr..

[24] Cerasani J., Ceroni F., De Cosmi V., Mazzocchi A., Morniroli D., Roggero P., Mosca F., Agostoni C., Gianni M.L. (2020). Human Milk Feeding and Preterm Infants’ Growth and Body Composition: A Literature Review. Nutrients.

[25] Lund A.M., Lofqvist C., Pivodic A., Lundgren P., Hard A.L., Hellstrom A., Hansen-Pupp I. (2020). Unpasteurised maternal breast milk is positively associated with growth outcomes in extremely preterm infants. Acta Paediatr..

[26] Picaud J.C., Buffin R. (2017). Human Milk-Treatment and Quality of Banked Human Milk. Clin. Perinatol..

[27] Ong K.K., Kennedy K., Castaneda-Gutierrez E., Forsyth S., Godfrey K.M., Koletzko B., Latulippe M.E., Ozanne S.E., Rueda R., Schoemaker M.H. (2015). Postnatal growth in preterm infants and later health outcomes: A systematic review. Acta Paediatr..

[28] Piemontese P., Mallardi D., Liotto N., Tabasso C., Menis C., Perrone M., Roggero P., Mosca F. (2019). Macronutrient content of pooled donor human milk before and after Holder pasteurization. BMC Pediatr..

[29] Zhang S.Q., Sproles A., Fu T.T. (2025). Effect of Pasteurization on Ghrelin and Resistin Hormone Concentrations in Human Breast Milk. Breastfeed. Med..

[30] Conboy-Stephenson R., Ross R.P., Kelly A.L., Stanton C. (2024). Donor human milk: The influence of processing technologies on its nutritional and microbial composition. Front. Nutr..

[31] Peila C., Moro G.E., Bertino E., Cavallarin L., Giribaldi M., Giuliani F., Cresi F., Coscia A. (2016). The Effect of Holder Pasteurization on Nutrients and Biologically-Active Components in Donor Human Milk: A Review. Nutrients.

[32] Munblit D., Treneva M., Peroni D.G., Colicino S., Chow L., Dissanayeke S., Abrol P., Sheth S., Pampura A., Boner A.L. (2016). Colostrum and Mature Human Milk of Women from London, Moscow, and Verona: Determinants of Immune Composition. Nutrients.

[33] Peroni D.G., Pescollderungg L., Piacentini G.L., Rigotti E., Maselli M., Watschinger K., Piazza M., Pigozzi R., Boner A.L. (2010). Immune regulatory cytokines in the milk of lactating women from farming and urban environments. Pediatr. Allergy Immunol..

[34] Ustundag B., Yilmaz E., Dogan Y., Akarsu S., Canatan H., Halifeoglu I., Cikim G., Aygun A.D. (2005). Levels of cytokines (IL-1beta, IL-2, IL-6, IL-8, TNF-alpha) and trace elements (Zn, Cu) in breast milk from mothers of preterm and term infants. Mediat. Inflamm..

[35] Kim S.Y., Yi D.Y. (2020). Components of human breast milk: From macronutrient to microbiome and microRNA. Clin. Exp. Pediatr..

[36] Richardson D.K., Corcoran J.D., Escobar G.J., Lee S.K. (2001). SNAP-II and SNAPPE-II: Simplified newborn illness severity and mortality risk scores. J. Pediatr..

[37] Fenton T.R., Kim J.H. (2013). A systematic review and meta-analysis to revise the Fenton growth chart for preterm infants. BMC Pediatr..

[38] Lederer D.J., Bell S.C., Smyth A.R., Chalmers J.D. (2019). Reply: More on Causal Inference Studies. Ann. Am. Thorac. Soc..

[39] Lederer D.J., Bell S.C., Branson R.D., Chalmers J.D., Marshall R., Maslove D.M., Ost D.E., Punjabi N.M., Schatz M., Smyth A.R. (2019). Control of Confounding and Reporting of Results in Causal Inference Studies. Guidance for Authors from Editors of Respiratory, Sleep, and Critical Care Journals. Ann. Am. Thorac. Soc..

[40] Morales Y., Schanler R.J. (2007). Human milk and clinical outcomes in VLBW infants: How compelling is the evidence of benefit?. Semin. Perinatol..

[41] Schanler R.J. (2011). Outcomes of human milk-fed premature infants. Semin. Perinatol..

[42] Schanler R.J. (2007). Mother’s own milk, donor human milk, and preterm formulas in the feeding of extremely premature infants. J. Pediatr. Gastroenterol. Nutr..

[43] Committee on Nutrition, Section on Breastfeeding, Committee on Fetus and Newborn (2017). Donor Human Milk for the High-Risk Infant: Preparation, Safety, and Usage Options in the United States. Pediatrics.

[44] Asbury M.R., Butcher J., Copeland J.K., Unger S., Bando N., Comelli E.M., Forte V., Kiss A., LeMay-Nedjelski L., Sherman P.M. (2020). Mothers of Preterm Infants Have Individualized Breast Milk Microbiota that Changes Temporally Based on Maternal Characteristics. Cell Host Microbe.

[45] Bancalari E. (2001). Changes in the pathogenesis and prevention of chronic lung disease of prematurity. Am. J. Perinatol..

[46] Maffei D., Schanler R.J. (2017). Human milk is the feeding strategy to prevent necrotizing enterocolitis!. Semin. Perinatol..

[47] Putz E., Ascherl R., Wendt T., Thome U.H., Gebauer C., Genuneit J., Siziba L.P. (2024). The association of different types of human milk with bronchopulmonary dysplasia in preterm infants. Front. Nutr..

[48] Miller J., Tonkin E., Damarell R.A., McPhee A.J., Suganuma M., Suganuma H., Middleton P.F., Makrides M., Collins C.T. (2018). A Systematic Review and Meta-Analysis of Human Milk Feeding and Morbidity in Very Low Birth Weight Infants. Nutrients.

[49] Sayed D., Abdellatif M. (2011). MicroRNAs in development and disease. Physiol. Rev..

[50] Underwood M.A. (2016). Missed Opportunities: The Cost of Suboptimal Breast Milk Feeding in the Neonatal Intensive Care Unit. J. Pediatr..

[51] Pineiro-Ramos J.D., Parra-Llorca A., Ten-Domenech I., Gormaz M., Ramon-Beltran A., Cernada M., Quintas G., Collado M.C., Kuligowski J., Vento M. (2021). Effect of donor human milk on host-gut microbiota and metabolic interactions in preterm infants. Clin. Nutr..

[52] Dicky O., Ehlinger V., Montjaux N., Gremmo-Feger G., Sizun J., Roze J.C., Arnaud C., Casper C., EPIPAGE 2 Nutrition Study Group, EPINUTRI Study Group (2017). Policy of feeding very preterm infants with their mother’s own fresh expressed milk was associated with a reduced risk of bronchopulmonary dysplasia. Acta Paediatr..

[53] Madore L.S., Sen S. (2017). Inconsistencies in Outcomes of Donor Breast Milk for Preterm Infants. Clin. Ther..

